# Changes in cardiac morphometry in fetuses with lower urinary tract obstruction

**DOI:** 10.1002/pmf2.70291

**Published:** 2026-04-08

**Authors:** Sarah Araji, Betul Yilmaz Furtun, Sara Al‐Haddad, Roopali Donepudi, Magdalena Sanz Cortes, Jessian Munoz, Cara Buskmiller, Brian Burnett, Michael A. Belfort, Michael Jochum, Ahmed A. Nassr

**Affiliations:** ^1^ Department of Obstetrics and Gynecology Division of Maternal‐Fetal Medicine Texas Children's Hospital Baylor College of Medicine Houston Texas USA; ^2^ Department of Molecular and Human Genetics Baylor College of Medicine Houston Texas USA; ^3^ Department of Pediatrics Division of Pediatric Cardiology Texas Children's Hospital Baylor College of Medicine Houston Texas USA

**Keywords:** cardiac findings, fetal echocardiogram, fetal lower urinary tract obstruction

## Abstract

**Objective:**

Fetal lower urinary tract obstruction (LUTO) is associated with significant perinatal morbidity due to oligohydramnios, pulmonary hypoplasia, and progressive renal dysfunction. In addition to these well‐known sequelae, LUTO has been linked to various cardiac abnormalities on prenatal imaging, including cardiomegaly; ventricular hypertrophy; pericardial effusion; and, in some cases, relatively small left‐sided cardiac structures. This study aimed to comprehensively characterize detailed fetal echocardiographic features in LUTO and compare them with gestational age (GA)‐matched controls, adjusting for estimated fetal weight (EFW), with an emphasis on quantitative cardiac dimensions.

**Methods:**

This retrospective cohort study included fetuses diagnosed with isolated LUTO who underwent at least one fetal echocardiogram at our institution from 2013 to 2025. Fetuses with known genetic abnormalities or additional major structural anomalies were excluded. Controls consisted of GA‐matched (1:1) fetuses with normal cardiac anatomy. Quantitative cardiac measurements were compared between groups and adjusted for EFW.

**Results:**

Among 117 LUTO fetuses who underwent at least one fetal echocardiogram, qualitative findings included pericardial effusion (29%), right ventricular hypertrophy (27%), and left ventricular hypertrophy (25%). Compared with controls, LUTO fetuses demonstrated significantly higher cardiothoracic ratio and smaller cardiac structures, including the mitral and tricuspid valves, aortic and pulmonary valves, and branch pulmonary arteries (all *p* < 0.01). These differences remained significant after adjustment for EFW.

**Conclusion:**

Fetuses with LUTO demonstrate global cardiac morphometric changes, affecting both the left‐ and right‐sided structures. These may reflect impaired diastolic filling or decreased venous return. Our findings highlight the potential value of serial fetal echocardiography particularly following fetal intervention for monitoring cardiac adaptation and informing perinatal counseling.

## 1 INTRODUCTION

Fetal lower urinary tract obstruction (LUTO) can lead to oligohydramnios, pulmonary hypoplasia, and ultimately renal failure. The condition has an incidence of 2.2:10,000 live births [1, 2], and is associated with significant morbidity and mortality [3]. LUTO is typically diagnosed during the second trimester of pregnancy. The most common cause in males is posterior urethral valves, followed by other etiologies such as urethral atresia and obstructing ureterocele. Additional entities that can present with similar sonographic findings include cloacal malformation, megacystis–microcolon–hypoperistalsis syndrome, and prune belly syndrome. Because of its early onset and the central role of the urinary system in amniotic fluid dynamics, LUTO can adversely affect the development of multiple organ systems.

LUTO is frequently associated with abnormal cardiac findings on prenatal imaging, including cardiomegaly, ventricular hypertrophy, and pericardial effusion [4, 5]. Additional reports have described small left‐sided cardiac structures [5] and decreased biventricular myocardial deformation leading to impaired cardiac function [6]. Proposed mechanisms include the impact of bladder distention leading to mechanical vascular compression as well as the contribution of renal dysfunction and vasoactive mediators to altered cardiac function [4, 5]. Pulmonary hypoplasia, a consequence of oligohydramnios, may further disrupt pulmonary blood flow and exacerbate cardiac dysfunction [5].

We hypothesize that impaired diastolic filling and reduced venous return contribute to physiologic changes that alter quantitative dimensions of both right‐ and left‐sided cardiac structures. Because blood flow is a key determinant of growth of cardiac structures, potential disruption in filling and venous return could lead to smaller cardiac structures.

This study aimed to characterize detailed fetal echocardiographic features in LUTO at a unique “one‐off” examination and compare them with gestational age (GA)‐matched controls, adjusting for estimated fetal weight (EFW), with an emphasis on quantitative cardiac dimensions.

## 2 METHODS

This study was approved by the Institutional Review Board (IRB#H‐37494). A retrospective chart review was performed for all LUTO cases evaluated at our institution between July 2013 and August 2025. Diagnosis of LUTO was based on prenatal ultrasound findings of a persistently enlarged fetal urinary bladder accompanied by features of bladder outlet obstruction, including hydronephrosis and/or hydroureters.

Inclusion criteria were (1) fetuses diagnosed with isolated LUTO who underwent at least one fetal echocardiogram at our institution between 2013 and 2025; (2) singleton or dichorionic–diamniotic twin gestations; (3) absence of other prenatally diagnosed major structural anomalies; and (4) no known chromosomal abnormalities, clinically relevant copy number variants, or single gene disorders. Exclusion criteria were monochorionic twin gestations, presence of other major fetal structural anomalies, or a known genetic diagnosis contributing to the LUTO phenotype.

All cases were evaluated at our institution with comprehensive ultrasound examination and multidisciplinary consultations. Nearest‐neighbor matching was performed using GA to generate a 1:1 matched control cohort for downstream comparisons. Controls fetuses had normal cardiac anatomy and function and were referred for evaluation due to a family history of congenital heart disease or conception via in vitro fertilization. All fetal echocardiograms were performed prior to any fetal intervention. Quantitative cardiac measurements were compared between groups and adjusted for EFW.

Fetal echocardiography was performed using standard methodology, including two‐dimensional imaging, color Doppler, and pulsed‐wave Doppler. Studies were conducted using an ACUSON Sequoia C512 or ACUSON S2000 (Siemens Healthcare Erlanger), a Philips EPIQ CVx system (Philips Medical Systems), or a Vivid E9machine (GE Healthcare). Measurements of the mitral valve (MV), tricuspid valve (TV), aortic valve (AV), and pulmonary valve (PV) annuli, as well as dimensions of the aorta, main pulmonary artery (MPA), and branch pulmonary arteries, were obtained using appropriate standard fetal echocardiographic planes as described in established guideline [7, 8, 9]. Qualitative parameters including the presence of pericardial effusion, TV regurgitation, and evidence of left (LV) or right ventricular (RV) hypertrophy were evaluated. The magnitude of valvular regurgitation was categorized as absent, mild, moderate, or severe based on the width and extension of the regurgitant jet. Cardiothoracic area (CTA) ratio was determined by measuring the cardiac four chamber view area in diastole and dividing it by the transverse thoracic area. Cardiomegaly was defined as a cardiac area to thoracic area ratio >0.35 on the four‐chamber view [9, 10, 11]. Ventricular wall thickness was evaluated at end‐diastole by M‐mode or two‐dimensional echocardiography and compared to normal values for GA. RV or LV hypertrophy was also assessed subjectively, and the degree of hypertrophy was described as either absent, mild, moderate, or severe [7, 10].

A chart review was performed to obtain relevant clinical information, including maternal and fetal clinical characteristics, qualitative and quantitative fetal echocardiographic findings, and additional data such as results from genetic evaluations.

Descriptive statistics including means, standard deviations, variances, and ranges were calculated are presented as mean ± SD or as *n* (%). Univariate comparisons of continuous variables were analyzed on the raw scale using Wilcoxon rank‐sum test. Missing EFW values were imputed using bag imputation methods to allow for downstream analysis. All analyses, summary tables, and data visualizations were conducted in R version 4.4.1.

## 3 RESULTS

A total of 117 fetuses with LUTO met the inclusion criteria. The mean GA at the time of the first echocardiogram was lower in the LUTO group compared with controls (21.84 ± 4.34 vs. 23.40 ± 3.07 weeks, *p* < 0.01). EFW was lower among LUTO fetuses (596 ± 460 g) compared with controls (665 ± 451 g, *p* = 0.01). In our cohort, cardiac findings were observed across bladder volumes ranging from 2 to 370 mL, although bladder size was not consistently reported.

*Qualitative echocardiographic findings* in fetuses with LUTO included pericardial effusion (29%), RV hypertrophy (27%), LV hypertrophy (25%), and tricuspid regurgitation (27%) as shown in Table 1. Among fetuses with tricuspid regurgitation, severity was categorized as trivial in eight (25%) cases, mild in 18 (56%), moderate in two (6%), and severe in four (12%).

**TABLE 1:** Qualitative echocardiographic findings in 117 fetuses with lower urinary tract obstruction.

Fetal echocardiographic findings	Value
Cardiomegaly	7/117 (6%)
LV hypertrophy	29/117 (25%)
RV hypertrophy	32/117 (27%)
Pericardial effusion	34/117 (29%)
Tricuspid regurgitation any	32/117 (27%)
Trivial	8 (25%)
Mild	18 (56%)
Moderate	2 (6%)
Severe	4 (12%)
Mitral regurgitation	6/117 (5%)
Pulmonary valve regurgitation	5/117 (4%)

*Note*: Data are given as *n* (%).

Abbreviations: LV, left ventricular; RV, Right ventricular.

*Quantitative cardiac measurements* differed significantly between groups (Table 2). Valve annuli were smaller in LUTO fetuses, including the AV (0.34 ± 0.10 cm vs. 0.37 ± 0.07 cm, *p* < 0.01), MV (0.56 ± 0.18 cm vs. 0.62 ± 0.12 cm, *p* < 0.01), PV (0.41 ± 0.13 cm vs. 0.46 ± 0.10 cm, *p* < 0.01), and TV (0.57 ± 0.20 cm vs. 0.64 ± 0.14 cm, *p* < 0.001; see Table 2).

**TABLE 2:** Clinical data and echocardiographic measurements of fetuses with lower urinary tract obstruction and gestational age‐matched normal controls adjusted for weight.

Variable	LUTO *(n =* 117)	Control *(n =* 117)	*p*
Gestational age (weeks)	21.84 ± 4.34	23.40 ± 3.07	<0.01
Estimated fetal weight (g)	596 ± 460	665 ± 451	0.01
Combined flow div weight (mL/min/kg)	497.98 ± 123.91	510.49 ± 59.51	0.09
LV cardiac output (mL/min)	127.87 ± 98.18	145.4 ± 89.64	0.07
RV cardiac output (mL/min)	179.69 ± 159.59	186.56 ± 127.39	0.63
CTA ratio	0.27 ± 0.06	0.23 ± 0.03	<0.01
Aortic measurements			
AV annulus D (cm)	0.34 ± 0.10	0.37 ± 0.07	<0.01
Aortic isthmus D (cm)	0.28 ± 0.08	0.29 ± 0.05	0.41
Ascending aorta D (cm)	0.42 ± 0.13	0.43 ± 0.08	0.22
Transverse arch D (cm)	0.35 ± 0.09	0.36 ± 0.06	0.3
LV measurements			
LV—LV long axis, D (cm)	1.55 ± 0.43	1.69 ± 0.31	0.01
LV—LV short axis, D (cm)	0.89 ± 0.28	0.88 ± 0.16	0.5
MV measurements			
MV annulus D (cm)	0.56 ± 0.18	0.62 ± 0.12	<0.01
PV and PA measurements			
PV annulus D (cm)	0.41 ± 0.13	0.46 ± 0.10	<0.01
PV and PA—LPA D (cm)	0.23 ± 0.08	0.25 ± 0.05	0.01
PV and PA—MPA D (cm)	0.47 ± 0.16	0.51 ± 0.11	0.01
PV and PA—RPA D (cm)	0.23 ± 0.07	0.26 ± 0.06	0.01
RV measurements			
RV—RV long Axis, D (cm)	1.41 ± 0.38	1.57 ± 0.31	0.01
RV—RV short Axis, D (cm)	0.85 ± 0.29	0.83 ± 0.15	0.62
TV measurements			
TV annulus D (cm)	0.57 ± 0.20	0.64 ± 0.14	<0.01

*Note*: Values are given as mean ± SD. EFW was unavailable in 19 fetuses but did not differ systematically between groups.

Abbreviations: AV, aortic valve; CTA ratio, cardiothoracic area ratio; D, diameter; div, divided; EFW, estimated fetal weight; LPA, left pulmonary artery; LV, left ventricle; MPA, main pulmonary artery; MV, mitral valve; PA, pulmonary artery; PV, pulmonic valve; RPA, right pulmonary artery; RV, right ventricle; TV, tricuspid valve.

Ventricular dimensions showed a similar pattern. The LV long‐axis dimension was significantly reduced in LUTO fetuses (1.55 ± 0.43 cm vs. 1.69 ± 0.31 cm, *p* = 0.01), as was the RV long‐axis dimension (1.41 ± 0.38 cm vs. 1.57 ± 0.31 cm, *p* = 0.01; see Table 2). Short‐axis dimensions of the ventricles, in contrast, did not differ significantly between groups.

The CTA ratio was markedly higher in the LUTO group compared with controls (0.27 ± 0.06 vs. 0.23 ± 0.03, *p* < 0.01), suggesting disproportionate changes in thoracic and cardiac dimensions. MPA and its branch dimensions also demonstrated group differences. The MPA diameter was smaller in LUTO fetuses (0.47 ± 0.16 cm vs. 0.51 ± 0.11 cm, *p* = 0.01), similarly the left pulmonary artery diameter was smaller in LUTO fetuses (0.23 ± 0.08 cm vs. 0.25 ± 0.05 cm, *p* = 0.01), as was the right pulmonary artery diameter (0.23 ± 0.07 cm vs. 0.26 ± 0.06 cm, *p* = 0.01; see Table 2).

No significant differences were observed in the aortic isthmus diameter, ascending aorta diameter, transverse arch diameter, or mitral/tricuspid inflow velocities, indicating that not all flow‐related parameters were altered. Likewise, LV and RV short‐axis diameters were not different between groups.

## 4 DISCUSSION

### 4.1 Principal findings

In our cohort, 25% of LUTO fetuses showed LV hypertrophy, 27% showed RV hypertrophy, 29% had pericardial effusion, and 27% demonstrated tricuspid regurgitation. Fetuses with LUTO demonstrated significantly higher CTA ratios and smaller valve annuli, ventricular long‐axis dimensions, and PAs compared to GA‐matched controls, even after adjusting for fetal weight.

### 4.2 Significance of findings

The higher CTA ratio in LUTO fetuses may reflect hemodynamic adaptation to altered loading conditions. Alternatively, apparent enlargement could represent relative cardiomegaly, related to a smaller chest diameter which can be secondary to compression of the thorax from severe oligohydramnios leading to pulmonary hypoplasia, as the absolute cardiac dimensions were, if anything, smaller rather than dilated. Our qualitative data further highlight the burden of cardiac structural and functional abnormalities in LUTO fetuses.

### 4.3 Prior studies

Our findings align with prior studies, which have similarly reported LV/RV hypertrophy and pericardial effusion [4] and have attributed these changes to increased afterload from iliac artery compression by the distended bladder.

The smaller left‐sided structures noted in our study in fetuses with LUTO, match findings from a prior study reporting smaller ascending aorta and aortic isthmus measurements in LUTO fetuses, with potential progression toward left‐heart hypoplasia [5]. That study also demonstrated an increased RV to LV cardiac index ratio and proposed that this reflects RV to LV cardiac output redistribution [5] secondary to LV diastolic dysfunction and reduced pulmonary blood flow from lung hypoplasia.

### 4.4 What our study adds

Our study identified smaller right‐sided structures, including smaller TV and PV annuli diameter, PA and its branches’ dimensions, and RV long axis dimension. Earlier work described abnormal RV filling in LUTO [11], with greater reliance on atrial contraction [4]. Those authors reported that the ductus venosus flow shows higher downstream impedance to filling leading to decreased RV compliance [4]. They hypothesized that because the RV is the major contributor to the cardiac output in fetal circulation, it may be particularly vulnerable to loading and hemodynamic changes. This observation is seen in other conditions such as twin to twin transfusion syndrome—where changes in filling and volume load are present—and is referred to as “right ventricle first and most” [4, 12, 13]. The authors’ main reported finding was RV hypertrophy; however, they did not describe smaller right‐sided cardiac structures, likely due to limited sample size and insufficient power to detect these differences. Our findings suggest that abnormal RV filling may ultimately lead to smaller right‐sided structures.

There may be additional explanations contributing to our right‐sided findings. Pulmonary hypoplasia leads to increased resistance in the pulmonary arteries [14]; moreover, upregulation of the renin–angiotensin–aldosterone (RAA) system, as discussed may drive both hypertrophy and abnormal diastolic filling leading to smaller right‐sided cardiac structures. Furthermore, direct mechanical compression by the distended bladder on iliac arteries leading to increase afterload [4] may also potentially cause compression on the IVC leading to decreased venous return to the right atrium and leading to impaired preload [15]. Notably, prior studies did not identify a direct relationship between bladder size and increased vascular impedance [4]. The presence of cardiac findings across a wide range of bladder volumes suggests that additional mechanisms are likely involved. One plausible explanation is activation of the fetal RAA system [15]. The upregulation of this system—which occurs because of renal parenchymal obliteration—increases afterload, contributing to biventricular hypertrophy and diastolic dysfunction [15, 16]. The high occurrence of TV regurgitation (27%) in our cohort further supports the presence of increased RV pressure and afterload [17].

### 4.5 Clinical implications

The findings of this study highlight the importance of systematic cardiac evaluation in fetuses diagnosed with LUTO. Beyond the urinary tract pathology, LUTO may be associated with significant cardiovascular remodeling, including both hypertrophic and relatively hypoplastic features, that may reflect altered fetal hemodynamics. Recognition of these patterns may provide additional insight into the cardiovascular effects of LUTO and could be useful in future studies aimed at risk stratification and perinatal management. Comprehensive fetal echocardiography should therefore be considered as part of the evaluation of LUTO cases at diagnosis and throughout surveillance, regardless of eligibility for fetal intervention.

Furthermore, these findings suggest that cardiac remodeling may evolve as a secondary phenomenon of increased afterload and altered preload, emphasizing the value of serial assessment to monitor progression. Prenatal cardiac findings may provide important prognostic information and help guide counseling. The coexistence of severe bladder outlet obstruction with cardiac remodeling may reflect advanced hemodynamic compromise and underscores the value of monitoring cardiovascular status throughout gestation. Postnatally, recognition of these cardiovascular alterations may highlight the importance of continued cardiac evaluation with attention to long‐term cardiovascular outcomes. Finally, the recognition that the RAA system may contribute to the observed remodeling provides a biological framework for potential targeted therapies or adjunctive monitoring strategies. Future studies integrating fetal cardiac indices with biomarkers of renal and cardiovascular function may refine risk stratification and improve outcomes in this complex population.

We recommend that pregnancies complicated by LUTO undergo at least one fetal echocardiogram regardless of intervention candidacy and be followed closely. The echocardiogram is also important to evaluate for structural cardiac abnormalities [18]. It is also recommended that delivery occurs at a tertiary care center capable of providing adequate cardiac evaluation and intervention as needed postnatally.

### 4.6 Strengths and limitations

To our knowledge, this study is the largest LUTO cohort with detailed echocardiographic findings across multiple cardiac structures. The rarity and high mortality of LUTO make this a valuable unique cohort. We excluded all cases with major structural and genetic abnormalities, although not all fetuses had genetic work up available. We adjusted for EFW even after GA‐matching controls. Limitations include the retrospective design preventing formal inter‐ and intra‐observer variability testing for qualitative measurements, variable completeness and unblinded assessment of echocardiographic findings, and inability to directly correlate findings with postnatal outcomes. Mechanistic pathways remain speculative, and prospective multicenter studies are needed to confirm these associations and assess longitudinal cardiac adaptation.

## 5 CONCLUSION

LUTO fetuses demonstrate global cardiac morphometric changes, affecting both the left and right‐sided structures. These findings likely reflect impaired diastolic filling and decreased venous return, compounded by vasoactive mediators’ effect. Our findings suggest that serial fetal echocardiography may provide additional insight into the evolution of these cardiac changes, although further evidence is needed to determine whether such monitoring would influence clinical management or outcomes. Further studies are needed to address the effect of the fetal cardiovascular findings on long‐term outcomes, survival, and to correlate them with postnatal echocardiographic findings.

## CONFLICT OF INTEREST STATEMENT

The authors declare no conflicts of interest.

## FUNDING INFORMATION

The authors received no specific funding for this work.

## Data Availability

The data that support the findings of this study are available on request from the corresponding author. The data are not publicly available due to privacy or ethical restrictions.
